# Morphogenetic Expression of Genes in Class II Division 2 Malocclusion: A Pedigree Study

**DOI:** 10.7759/cureus.83647

**Published:** 2025-05-07

**Authors:** Sagar Hirani, Tanvi Hirani, Alap Shah, Shirishkumar Patel, Miral Mehta, Pratiksha Patel, Santosh Kumar, Mainul Haque

**Affiliations:** 1 Department of Periodontology and Implantology, College of Dental Science and Research Centre, Gujarat University, Ahmedabad, IND; 2 Department of Periodontology and Implantology, Karnavati School of Dentistry, Karnavati University, Gandhinagar, IND; 3 Department of Orthodontics and Dentofacial Orthopedics, Karnavati School of Dentistry, Karnavati University, Gandhinagar, IND; 4 Department of Pedodontics and Preventive Dentistry, Karnavati School of Dentistry, Karnavati University, Gandhinagar, IND; 5 Department of Pharmacology and Therapeutics, National Defence University of Malaysia, Kuala Lumpur, MYS; 6 Department of Research, Karnavati School of Dentistry, Karnavati University, Gandhinagar, IND

**Keywords:** class ii division 2, craniofacial deformities, familial dentofacial, genetics, gregor mendel, hereditary factors, hereditary transmission, malocclusion, orthodontics, pedigree study

## Abstract

Introduction

The etiology of malocclusion is multifactorial, involving both genetic and environmental influences. The literature has shown that neither a single entity, hereditary or genetic factors, nor ecological factors alone are responsible for causing malocclusion; furthermore, constant thumb-sucking and pacifier use, bony anomalies, congenitally missing teeth, oral injury, mouth breathing, etc., often cause malocclusion. Participants were selected from those who reported to the outpatient department of the Karnavati School of Dentistry in Gandhinagar, Gujarat, India, indicating a clinic-based sampling method. Both hereditary and environmental factors were equally considered as causative factors. This research aims to determine how craniofacial patterns associated with class II division 2 malocclusion are inherited from parents to their children through a pedigree analysis.

Methods

Patients diagnosed with class II division 2 malocclusion underwent thorough intraoral and extraoral assessments. Cephalometric tracings were included in the study after meeting the established inclusion and exclusion criteria. Family trees were created and analyzed using Cyrillic Software.

Results

A stronger correlation was found between patients and their fathers regarding skeletal and dental measurements. In contrast, the correlation coefficients for skeletal parameters between patient-mother pairs showed highly significant correlations, whereas no statistically significant correlation was found for dental parameters.

Conclusion

The morphogenetic expression in this study revealed that class II division 2 malocclusion exhibited sexual dimorphism for this specific category. Genetic counseling for parents can aid in early diagnosis and the development of prevention strategies. Mutations that are likely to occur due to the presence or absence of a particular gene or group of genes can be avoided if the prevalence of the specific gene associated with that condition is known.

## Introduction

The etiology of malocclusion remains unknown [1,2]. This multifactorial etiology implies that, while the causes are partially understood, the relative contribution of each factor remains unknown [3,4]. The literature indicates that neither hereditary genetics nor ecological factors alone are responsible for causing malocclusion, further suggesting that it can have a multifactorial origin with both factors being equally causative [5].

Gregor Mendel's groundbreaking research in the 19th century sparked a fascination with genetics, establishing it as a crucial area of study in biological and medical research ever since [6,7]. The causes of malocclusion are diverse, with genetic and environmental influences significantly contributing to its onset [8]. Genetics and environmental influences are recognized as significant contributors to the development of skeletal anomalies [9].

Diagnosing familial dentofacial issues considers genetics a key factor influencing the development of malocclusion [10]. The clinician must deeply understand recent developments in genetics and their relevance to orthodontics [11]. Recent research and progress in genetic science have enabled orthodontists to gain deeper insights into how genetics influences the causes of dentofacial traits and disorders [12-14]. This understanding has further reinforced the role of genes in forming the dentofacial complex [15].

When addressing orthodontic cases, it is essential to consider the impact of genetics on diagnosing skeletal anomalies [5,16]. By identifying and distinguishing hereditary factors, the clinician can effectively assess the environmental influences and develop a targeted treatment plan [17]. Class II division 2 malocclusion is a polygenic condition that arises from genetic predispositions and environmental influences [18,19].

This study aimed to assess the hereditary transmission of craniofacial patterns associated with class II division 2 malocclusion from parents to their children via a pedigree analysis and evaluate the degree of phenotypic expression of class II division 2 malocclusion in the offspring.

## Materials and methods

**Figure 1:**
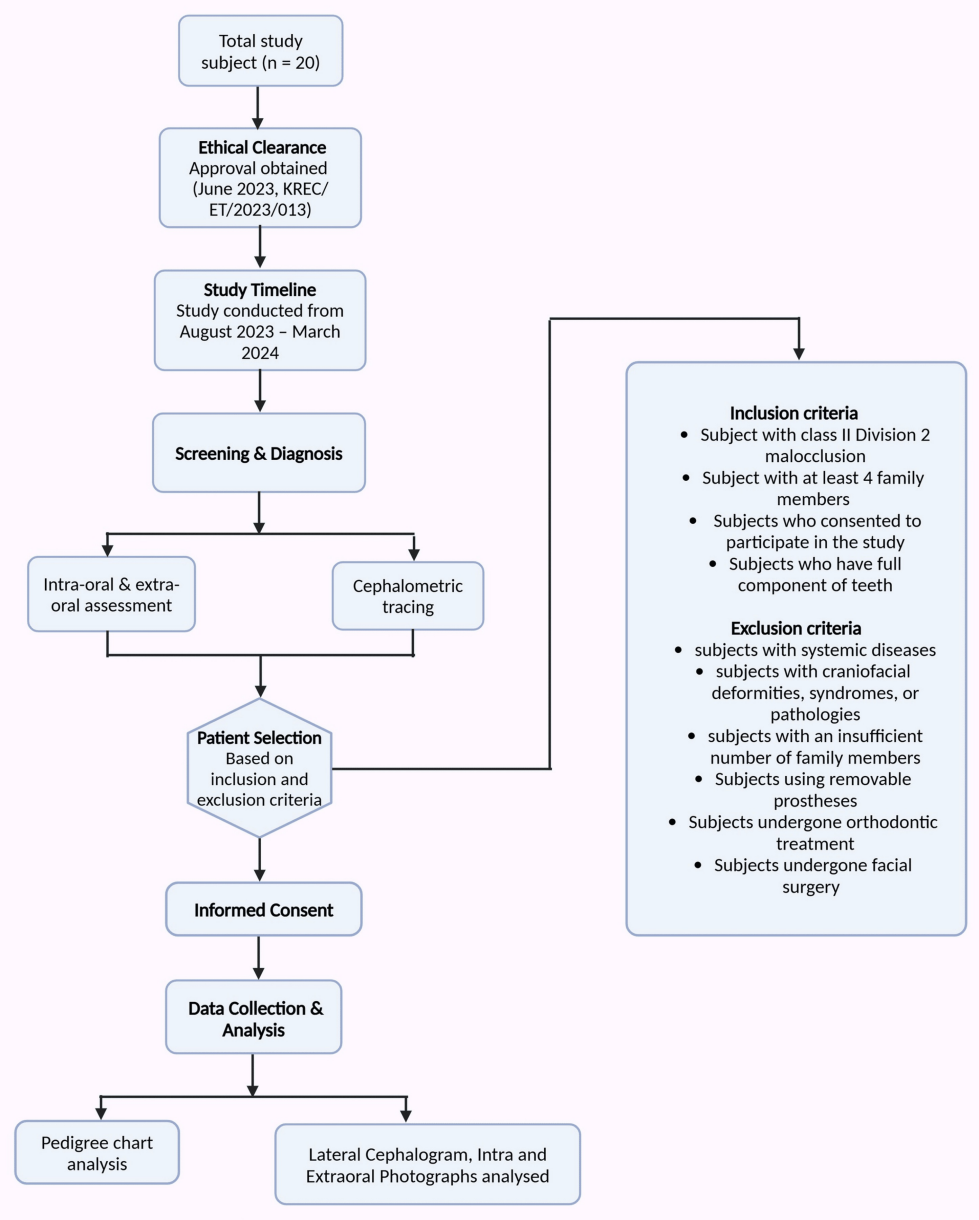
Methodology of the study Notes: This image was created using the premium version of BioRender (https://BioRender.com/wwqfbcd) [20] accessed on March 23, 2025, with agreement license number Missing KJ2825INP0. We did not utilize anything from the BioRender Template library. Illustration credit: Pratiksha Patel

Research participants were selected from the outpatient clinic of the Department of Orthodontics and Dentofacial Orthopedics at Karnavati School of Dentistry in India. These patients were initially screened for a class II malocclusion through intraoral and extraoral examinations, as well as cephalometric tracing. Research participants were selected based on age, and universal sampling was adopted. All patients having class II division 2 malocclusion were included in our study. The current study was conducted from August 2023 to March 2024. Twenty patients with a class II division 2 malocclusion were identified from the screening sample and included in the study, provided they met the inclusion and exclusion criteria. Informed consent was obtained from the probands after explaining the nature and further scope of the study. The pedigree chart was portrayed and analyzed using cephalometric analyses, which were performed using Dolphin Imaging Software version 11.9 (Figure 1). The study was conducted at a 95% confidence level for statistical significance.

Furthermore, subjects with insufficient family members were excluded, as it was impossible to accurately establish pedigree charts and genetic correlations. The study also excluded individuals using removable prostheses, as their dental malocclusion patterns could not be assessed appropriately. Those who had undergone orthodontic treatment were omitted, as their malocclusion could not be reliably compared to their parental origin. Similarly, individuals who had undergone facial surgery were excluded to prevent misinterpretation of the malocclusion diagnosis. Lateral cephalograms and intra- and extraoral photographs were documented and analyzed for all subjects involved in this study: participants with probands, their parents, and siblings.

Skeletal diagnosis

Linear and angular hard and soft tissue measurements determined skeletal, dental, and facial types. Steiner, McNamara, Downs, Wits AO-BO, and Jarabak ratios were considered for the cephalometric interpretation of malocclusion. The following cephalometric parameters were taken into consideration to compare and correlate the extent of their deviation among the parents and offspring: Sella-Nasion-A (SNA), Sella-Nasion-B (SNB), anteroposterior relationship between the maxilla and mandible (ANB), posterior facial height (PFH)/anterior facial height (AFH) ratio, Y-axis, angle of convexity, A perpendicular Nasion (A┴N), Pogonion perpendicular Nasion (Pg┴N), Sella-Nasion/Mandibular Plane (SN-MP), maxillary base length (Co-A), mandibular base length (Co-Gn), saddle angle (N-S-Ar), articular angle (S-Ar-Go), and gonial angle (Ar-Go-Me).

Dental diagnosis

Dental diagnoses were conducted using study models: a 3Shape scanner (3Shape A/S, Copenhagen, Denmark ) and orthoanalyzer version 1.6 (TeselaGen Biotechnology Inc., San Francisco, CA) were utilized for model acquisition. The malocclusion classification of all participants was determined by analyzing lateral cephalograms and dental models. Angles and Profitt's classification systems for malocclusion were considered for dental diagnosis [21]. The following dental cephalometric parameters were considered to compare and correlate the extent of their deviation among the parents and offspring: U1-SN (angle between the long axis of the most protruded maxillary incisor (U1) and the Sella-Nasion (SN) line), U1-L1 (angle between the long axis of the upper incisors (U1) and the long axis of the lower incisors (L1)), and L1-MP (angle between the long axis of the lower central incisor (L1) and the mandibular plane (MP)).

Soft tissue diagnosis

All participants' standardized frontal and lateral facial photographs, including their parents and siblings, were captured and examined for similarities and differences. To be eligible for inclusion in the study, all participants had to exhibit confirmed positive ANB angles, a straight or convex profile, and a deep bite.

Ethical approval****

Ethical approval was obtained from the Institutional Review Board (IRB) of Karnavati School of Dentistry and Hospital, Karnavati University, Gandhinagar, Gujarat, India, on May 13, 2024 (KSDEC/2024/Apr/008). The study followed the protocol as per the Helsinki Declaration. The research participants were verbally explained the research plan and future publication. Additionally, we have ensured that participants of the current study are aware of the anonymity of research and publication.

The study included subjects diagnosed with class II division 2 malocclusion, both skeletal and dental. Only individuals with at least four family members were considered to ensure the accuracy of the pedigree analysis. Additionally, participants were required to have a complete set of teeth and be willing to participate in the study. However, subjects with systemic diseases, such as endocrine and connective tissue disorders, were excluded, possibly influencing the results. Individuals with craniofacial deformities, syndromes, or pathologies were also not considered to avoid false interpretations.

## Results

Table 1 shows the study participants, their mean age, and the standard deviation.

**Table 1:** Study samples and their mean age Table credit: Sagar Hirani

Variables	Number of patients	Age (years)
Patient	20	12 ± 3.5
Father	20	53 ± 3.5
Mother	20	50 ± 2.5

Table 2 presents the mean and standard deviation values of skeletal and dental measurements for probands and their parents. Pearson's correlation coefficient was calculated to assess the degree of similarity and inheritance of these features from parents to offspring. Additionally, Table 3 provides the computed Pearson's correlation coefficient values, illustrating the relationship between the father and the patient and between the mother and the patient for the specified skeletal and dental parameters listed in the first column.

**Table 2:** Mean and standard deviation values of the parametric measurement Notes: SNA: Sella-Nasion-A point angle, SNB: Sella-Nasion-B point, ANB: angle's anteroposterior relationship between the maxilla and mandible angle, N┴Point A: Nasion-Point A measurement, N┴Pogonion: Pogonion to Nasion perpendicular, PFH/AFH ratio: posterior facial height-to-anterior facial height ratio, SN-MP: Sella-Nasion/Mandibular Plane angle, N-S-Ar: ratio to the saddle angle, S-Ar-Go: articular angle, which is measured between the sella (S), articulare (Ar), and gonion (Go) points on a lateral cephalogram, Ar-Go-Me: Ar-Go-Me angle, U1-SN: angle between the long axis of the most protruded maxillary incisor (U1) and the Sella-Nasion (SN) line, L1-MP: angle between the long axis of the lower central incisor (L1) and the mandibular plane (MP), a measurement used in cephalometric analysis to assess tooth position and skeletal relationships, U1-L1: angle between the long axis of the upper incisors (U1) and the long axis of the lower incisors (L1), a measurement used in cephalometric analysis Table credit: Sagar Hirani

Parameters			Patient (measurements in MM)	Father (measurements in MM)	Mother (measurements in MM)
Skeletal					
SNA	Position of the maxilla about the cranial base		83.8 ± 1.36	83.9 ± 1.48	83.6 ± 1.84
SNB	Position of the mandible to the cranial base		78.4 ± 1.46	80.1 ± 2.48	78.5 ± 1.73
ANB	Relation of the maxilla to the mandible		5.4 ± 0.82	3.8 ± 1.64	5.1 ± 0.96
N┴Point A	Maxilla position		2.5 ± 2.35	2.7 ± 1.12	2.5 ± 1.23
N┴Pogonion	Mandible position		-0.1 ± 6.16	1.5 ± 3.25	1.5 ± 2.98
Condylion-Point A	Effective maxillary length		88 ± 5.23	84.9 ± 2.53	85.2 ± 5.63
Condylion-Gnathion	Effective mandibular length		103.6 ± 5.39	105.2 ± 5.81	102.4 ± 4.59
PFH/AFH ratio	Growth pattern		69.5 ± 6.5	70 ± 4.23	66.85 ± 5.38
Y-axis	Growth pattern		61.5 ± 3.2	65 ± 6.6	60.1 ± 2.6
SN-MP	Growth pattern		25.6 ± 3.43	24.6 ± 2.39	26 ± 1.12
Angle of convexity	Profile		4.1 ± 2.07	1.3 ± 4.9	2.0 ± 3.4
N-S-Ar	Position of the mandible about the cranial base		125.8 ± 6.35	124.5 ± 7.0	124.9 ± 7.99
S-Ar-Go	Position of the mandible about the cranial base		139.2 ± 5.65	139.5 ± 3.15	139.8 ± 4.42
Ar-Go-Me	Mandibular rotation		121.5 ± 4.5	119.3 ± 3.8	122.1 ± 2.73
Dental					
U_1_-SN		Inclination of the incisors to the cranial base	87.6 ± 2.7	87.2 ± 2.93	93.6 ± 9.0
L_1_-MP		Inclination of the incisors in relation to the mandibular plane	87.6 ± 1.53	87.5 ± 2.25	91.4 ± 6.03
U_1_-L_1_		Interincisal angle	168.4 ± 3.76	167.6 ± 4.15	150.2 ± 15.6

**Table 3:** Pearson's correlation coefficients for the measurements taken from both patients and their parents Notes: *p < 0.05, **p < 0.01 Notes: SNA: Sella-Nasion-A point angle, SNB: Sella-Nasion-B point, ANB: angle's anteroposterior relationship between the maxilla and mandible angle, N┴Point A: Nasion-Point A measurement, N┴Pogonion: Pogonion to Nasion perpendicular, PFH/AFH ratio: posterior facial height-to-anterior facial height ratio, SN-MP: Sella-Nasion/Mandibular Plane angle, N-S-Ar: ratio to the saddle angle, S-Ar-Go: articular angle, which is measured between the sella (S), articulare (Ar), and gonion (Go) points on a lateral cephalogram, Ar-Go-Me: Ar-Go-Me angle, U1-SN: angle between the long axis of the most protruded maxillary incisor (U1) and the Sella-Nasion (SN) line, L1-MP: angle between the long axis of the lower central incisor (L1) and the mandibular plane (MP), a measurement used in cephalometric analysis to assess tooth position and skeletal relationships, U1-L1: angle between the long axis of the upper incisors (U1) and the long axis of the lower incisors (L1), a measurement used in cephalometric analysis. Table credit: Sagar Hirani

Parameters		Patient x father	Patient x mother
Skeletal			
SNA	Position of the maxilla in relation to the cranial base	-0.063	0.511*
SNB	Position of the mandible in relation to the cranial base	-0.589**	0.747**
ANB	Relation of the maxilla to the mandible	-0.016	0.079
N┴Point A	Maxilla position	-0.337	-0.091
N┴Pogonion	Mandible position	0.207	0.100
Condylion-Point A	Effective maxillary length	0.000	0.728**
Condylion-Gnathion	Effective mandibular length	-0.007	0.814**
PFH/AFH ratio	Growth pattern	0.333	0.678**
Y-axis	Growth pattern	0.183	0.006
SN-MP	Growth pattern	0.619**	-0.218
Angle of convexity	Profile	0.131	0.641**
N-S-Ar	Position of the mandible in relation to the cranial base	0.788**	0.777**
S-Ar-Go	Position of the mandible in relation to the cranial base	0.325	0.579**
Ar-Go-Me	Mandibular rotation	0.629**	-0.364
Dental			
U_1_-SN	Inclination of the incisors in relation to the cranial base	0.763**	0.349
L_1_-MP	Inclination of the incisors in relation to the mandibular plane	0.334	-0.175
U_1_-L_1_	Interincisal angle	0.576**	0.197

A stronger correlation was observed between patients and their parents regarding skeletal and dental measurements. In the analysis of skeletal measurements between patient-father pairs, a highly significant correlation (p < 0.01) was observed for SN-MP (growth pattern), N-S-Ar (mandible position relative to the cranial base), and Ar-Go-Me (mandibular rotation). Additionally, for dental measurements, significant correlations were found for U1-SN (incisor inclination concerning the cranial base) and U1-L1 (interincisal angle).

In contrast, the correlation coefficients for skeletal parameters between patient-mother pairs demonstrated a highly significant correlation for SNA (maxilla position relative to the cranial base), SNB (mandible position relative to the cranial base), Co-A (effective maxillary length), Co-Gn (effective mandibular length), AFH/PFH ratio (growth pattern), angle of convexity, N-S-Ar (mandible position relative to the cranial base), and S-Ar-Go (mandible position relative to the cranial base) (p < 0.01). However, no statistically significant correlation was observed for dental parameters.

## Discussion

Inheritance of the maxilla and mandible

**Figure 2:**
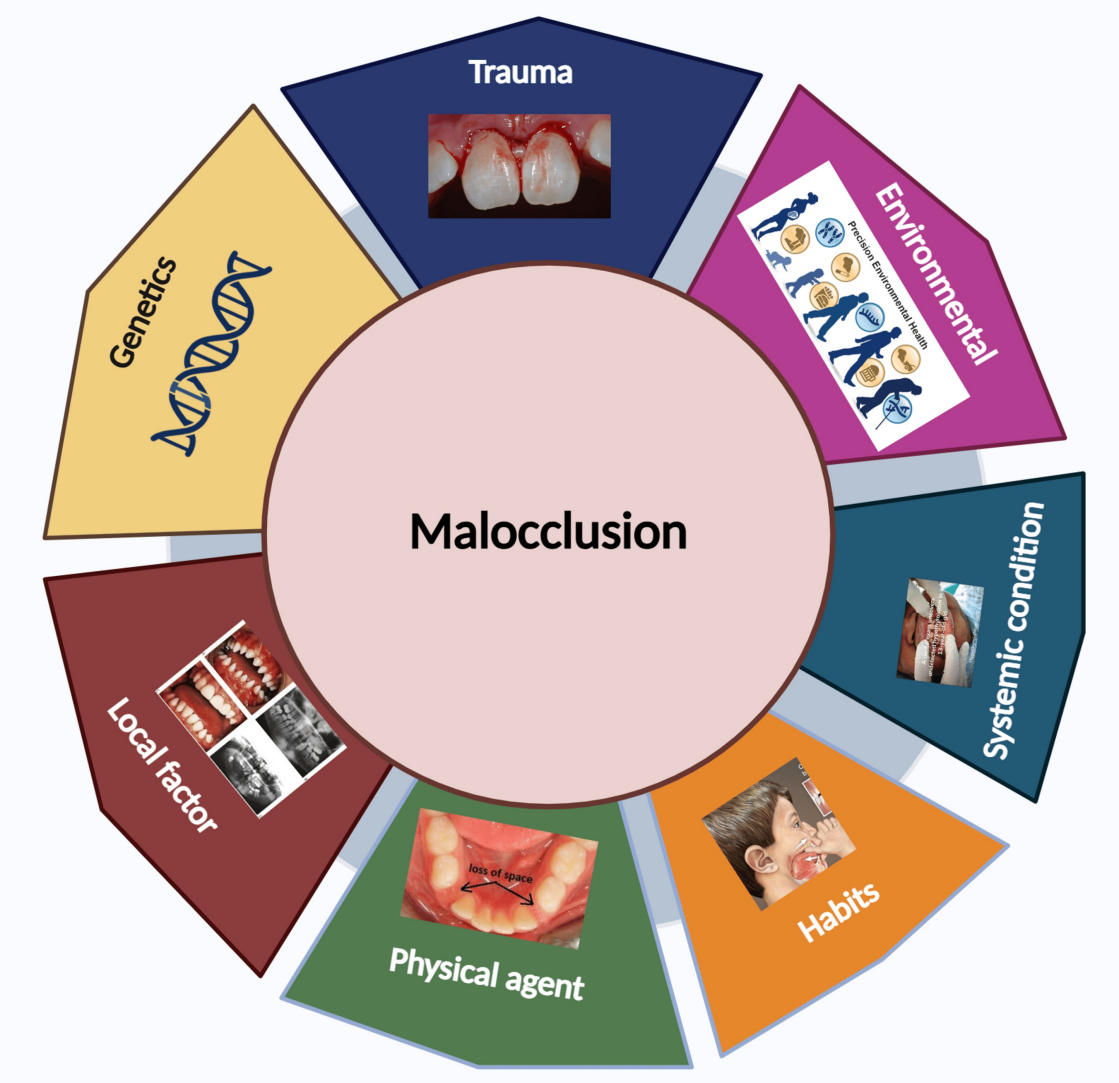
Illustration of the etiology of malocclusion Notes: This illustration was developed using the premium version of BioRender (https://BioRender.com/p4d7io0) [20] with an agreement license number ZC2826KAJG, accessed on March 23, 2025. We did not utilize anything from the BioRender Template Library. Illustration credit: Pratiksha Patel

The position of the maxilla, determined by SNA angle and Condylion-Point A, and that of the mandible, determined by SNB angle and Condylion-Point B, suggested a significant level of inheritance from mother to offspring compared to father to offspring. However, specific cephalometric parameters, such as N-perpendicular and N-perpendicular to Pogonion, showed no statistically significant correlation. Multiple earlier studies suggested that class II tendencies occur in Caucasians because their anterior cranial fossa is horizontally long and narrow [22-25]. The etiology of malocclusion is illustrated in Figure 2.

Inheritance of the rotation of jaw bases

Rotation of jaw bases evaluated from cephalometric parameters N-S-Ar (saddle angle), S-Ar-Go (articulare angle), and Ar-Go-Me (gonial angle) also showed inheritance pattern from mother to offspring and also from father to offspring, suggesting that the cephalometric correlations has possibility of patterns of inheritance but do not directly confirm genetic distribution. However, an adequate sample size must be considered to establish this fact. Giuntini et al. [26] and Kim et al. [27] have proposed that cephalometric analysis can be used to predict craniofacial growth based on the premise that the facial type of a growing child with malocclusion provides insight into the anticipated changes in craniofacial growth direction. The genetic foundation for this predictive approach is, regrettably, nearly nonexistent. Nevertheless, the accuracy of growth predictions can be improved by considering the craniofacial morphology of the parents [28,29].

Inheritance of growth pattern

**Figure 3:**
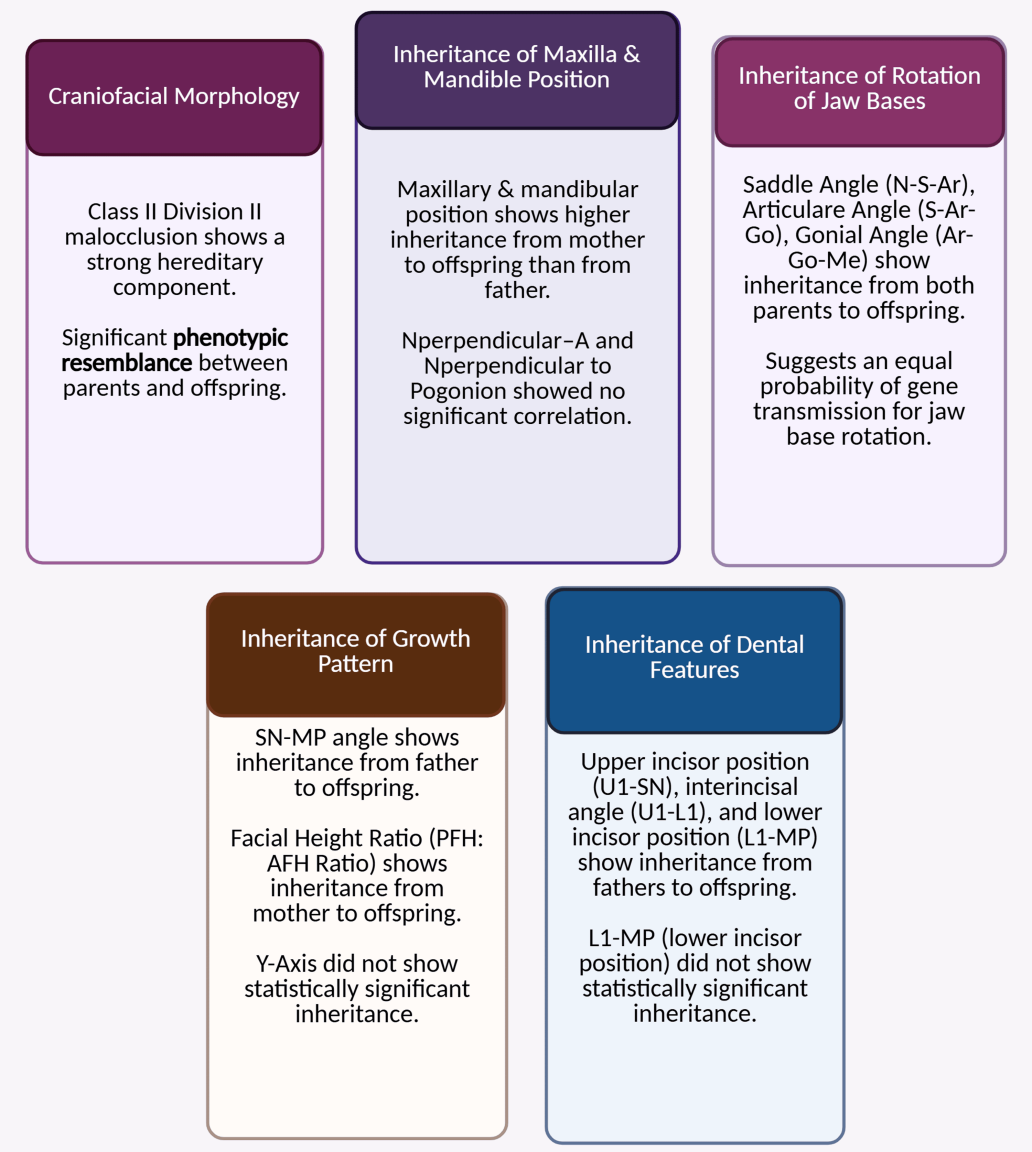
Principal findings of the paper Notes: This illustration was developed using the premium version of BioRender (https://BioRender.com/3ilcw8d) [20] with an agreement license number YU2825XT17, accessed on March 23, 2025. We did not utilize anything from the BioRender Template Library. Bold information denotes statistical significance. Notes: PFH/AFH ratio: posterior facial height-to-anterior facial height ratio, SN-MP: Sella-Nasion/Mandibular Plane angle, N-S-Ar: saddle angle, S-Ar-Go: articular angle, which is measured between the sella (S), articulare (Ar), and gonion (Go) points on a lateral cephalogram, Ar-Go-Me: Ar-Go-Me angle, U1-SN: angle between the long axis of the most protruded maxillary incisor (U1) and the Sella-Nasion (SN) line, L1-MP: angle between the long axis of the lower central incisor (L1) and the mandibular plane (MP), a measurement used in cephalometric analysis to assess tooth position and skeletal relationships, U1-L1: angle between the long axis of the upper incisors (U1) and the long axis of the lower incisors (L1), a measurement used in cephalometric analysis. Illustration credit: Pratiksha Patel

Growth pattern evaluated by cephalometric parameter (Y-axis, SN-MP, and facial height ratio (PFH/AFH ratio)) [30] suggested that there is a familial tendency of transmitting factors responsible for growth pattern from either parent to offspring, where the Y-axis did not show any statistically significant results. In contrast, SN-MP showed a substantial correlation between father and offspring, and the facial height ratio (PFH/AFH ratio) showed a statistically significant correlation between mother and offspring. The key findings of this are illustrated in Figure 3.

Inheritance of dental features

Dental characteristics (U1-SN, interincisal, and L1-MP) demonstrated a pattern of inheritance from fathers to their children, except the position of the lower incisor (L1-MP), which did not exhibit a statistically significant level of inheritance from parents to offspring. Given the limitations of cephalometric analysis in examining craniofacial morphology, it is essential to explore it using three-dimensional analysis. This approach enables identifying how these structures are inherited across various malocclusions. The distinction between genetic and environmental influences on the development of morphologic characteristics associated with class II division 2 malocclusion can significantly affect orthodontic treatment strategies, as areas influenced by ecological factors may be improved through orthodontic intervention [1,31]. However, malocclusion with a genetic etiology must be identified before treatment, as deformities that develop due to genes can sometimes require surgical intervention [3].

Limitations of the study

The study is limited by its small sample size and lack of genetic testing. There is a marked resemblance of phenotype between parents and offspring. Further, skeletal and dental features demonstrate different levels of inheritance from parent to offspring. Further, skeletal and dental features demonstrate different levels of inheritance from parent to offspring. Another limitation was that all cephalometric analyses were based on two-dimensional lateral cephalograms. We cannot address these issues as this study received no financial support.

Future scope of the study

This study highlights that malocclusion can have a multifactorial etiology. To better understand its genetic inheritance, specific genetic testing for particular genes or gene groups can be conducted to determine their specificity and reliability in contributing to the development of certain types of malocclusion. Additionally, a multicenter study with an adequate sample size had the potential to validate the role of inheritance in transmitting malocclusion traits. The study also highlights the importance of pedigree analysis in understanding hereditary malocclusion patterns. Furthermore, a large cohort of high-risk individuals could provide valuable insights and reinforce the role of genetic inheritance in the development of malocclusion across generations. To enhance accuracy in evaluating skeletal, dental, and facial features, three-dimensional imaging techniques such as cone-beam computed tomography (CBCT) can contribute to more precise statistical analysis and results.

## Conclusions

The morphogenetic expression observed in this study indicated that class II division 2 malocclusion exhibited sexual dimorphism within this specific category. Additionally, the phenotypic expression of certain probands closely resembled that of their parents, siblings, or affected relatives, suggesting that genetic factors probably play a significant role in the development of malocclusion. This underscores the significance of considering genetic predisposition when developing or implementing individual treatment plans. The level of penetrance and expressivity of the affected gene influences the severity of genotypic expression from parents to offspring. Further multicenter studies with a multicenter and large sample size and genetic analysis are necessary to validate this observation. Furthermore, genetic mutation studies regarding dental malocclusion should take on preventive measures to determine whether specific genes contribute to this issue.
